# How to assess clinical implication of catheter ablation for ventricular tachycardia in patients with structural heart diseases

**DOI:** 10.1002/joa3.70064

**Published:** 2025-04-11

**Authors:** Sayaka Matsumoto, Naoya Kataoka, Teruhiko Imamura

**Affiliations:** ^1^ Second Department of Internal Medicine University of Toyama Toyama Japan

To editor,

Catheter ablation for ventricular tachycardia (VT) in patients with structural heart disease has increasingly become prevalent, primarily due to advancements in functional substrate mapping techniques. However, clinicians have expressed concern regarding potential procedure‐related declines in cardiac function consequent to myocardial injury. The authors demonstrated that left ventricular ejection fraction (LVEF) remained preserved in the majority of patients postablation^1^; nonetheless, several pertinent issues warrant consideration.

The PAINESD score serves as a valuable tool for stratifying the risk of ablation‐related complications, including deterioration of cardiac function and the development of heart failure, with an LVEF of less than 25% constituting one of the key criteria.^2^ In the current study, nearly half of the subjects exhibited preserved LVEF at baseline.^1^ Consequently, any minor procedural reductions in LVEF might exert minimal clinical significance within this specific subgroup. Refining their findings by excluding patients with preserved LVEF could potentially enhance the interpretability and applicability of the outcomes.

Numerous participants received comprehensive pharmacotherapy, commonly referred to as the “fantastic four” regimen.^1^ Approximately half of the participants presented with preserved LVEF. However, robust evidence supporting the necessity and efficacy of these medications in this particular population remains limited.^3^ Notably, the present study identified the administration of renin‐angiotensin system inhibitors as a risk factor associated with reduced cardiac function following ablation. Baseline LVEF levels may represent a confounding variable influencing this observation.

The clinical significance of observed improvements in LVEF among patients already exhibiting preserved ejection fraction remains ambiguous. Evaluating the reduction in low‐voltage myocardial areas during postablation follow‐up could provide critical insights into the genuine impact of catheter ablation procedures in this subset of patients.

Finally, delineating the optimal area for ablation remains a significant technical challenge. Recent literature indicates the length of VT isthmus averages approximately 17 mm,^4^ typically necessitating only four to five ablation lesions using standard radiofrequency catheters. Such minimally invasive catheter ablation techniques theoretically minimize detrimental effects on cardiac function. Future research endeavors should focus on advancing mapping methodologies to precisely define optimal ablation targets, thereby potentially mitigating adverse effects on cardiac function.^5^

## CONFLICT OF INTEREST STATEMENT

Authors declare no conflict of interests for this article.

## Data Availability

The manuscript does not include any original data.
